# Efficacy and safety of massage therapy for autism spectrum disorders: A protocol for systematic review and meta-analysis

**DOI:** 10.1097/MD.0000000000025874

**Published:** 2021-05-14

**Authors:** Sheng Guo, Ke-Lin Zhou, Shuo Dong, Xiao-Na Xue, Pei-Dong Wei, Jing-Yi Yang, Guo-Bing Fu, Zhen-Bo Liu, Xia Cui

**Affiliations:** a Dongfang Hospital of Beijing University of Chinese Medicine; b Beijing University of Chinese Medicine; c The Third Affiliated Hospital of Beijing University of Chinese Medicine, Beijing, China.

**Keywords:** autism spectrum disorders, complementary and alternative medicine, massage therapy, protocol

## Abstract

**Background:**

Autism spectrum disorder is a neurodevelopmental disorder with increasing incidence. At present, the global incidence of the disease is on the rise, and the cause is unknown. There is no specific treatment for this disease at present, mainly education and training. Traditional Chinese medicine treatment has a certain effect on the improvement of the symptoms of the disease. The treatment methods are mainly oral Chinese medicine and acupuncture, but children are often not easy to cooperate. As a safe and effective green therapy, massage is easy to be accepted by children.

**Methods:**

We will search the following electronic databases for randomized controlled trials to evaluate the effectiveness and safety of massage therapy in treating autism spectrum disorders: Wanfang and PubMed Database, China National Knowledge Infrastructure Database, Cochrane Central Register of Controlled Trials, Cumulative Index of Nursing and Allied Health Literature, and Excerpta Medica database. Each database will be searched from inception to March 2021. The entire process will include study selection, data extraction, risk of bias assessment, and meta-analyses.

**Results:**

This proposed study will evaluate the effectiveness and safety of massage therapy for patients with autism spectrum disorders. The outcomes will include changes in autism spectrum disorder relief and adverse effect.

**Conclusion:**

This proposed systematic review will evaluate the existing evidence on the effectiveness and safety of massage therapy for patients with autism spectrum disorders.

**Dissemination and ethics:**

The results of this review will be disseminated through peer-reviewed publication. Because all of the data used in this systematic review and meta-analysis have been published, this review does not require ethical approval. Furthermore, all data will be analyzed anonymously during the review process.

## 1 Introduction

Autism spectrum disorder (ASD) is also called autism. The disease is a complex neurodevelopmental disorder with the core symptoms of social interaction disorder, narrow interest, and repetitive behavioral stereotypes.^[1]^ At present, the global incidence of the disease is on the rise, and the cause is unknown. According to data, the global prevalence of ASD in 2000 was about 1/160,^[2]^ and so far, about 1% of the world's population has ASD, and the ratio of male to female is 2.5:1.^[3,4]^ Data released by the US Centers for Disease Control and Prevention shows that in 2005, the incidence of autism in children was 1 in 166, while in 2018, it rose to 1 in 59, and the annual cost of children with ASD in the United States was as high as $130 billion.^[5–8]^ With the development of science and technology, the research on the etiology of ASD has been deepening gradually. Initially, the understanding of the etiology of ASD was limited to emotional disorders, attributed to improper parenting. And then he moved on to understanding the nature of the neurobiology, which came down to pervasive developmental disorders. At present, the pathogenesis of ASD is not clear, mainly including genetic factors,^[9–11]^ maternal and perinatal factors,^[12–17]^ immune factors,^[18–20]^ and neurological factors.^[21–24]^ However, most scholars believe that the occurrence of ASD is closely related to genetic factors, environmental factors, and their interaction.^[25]^ In addition, literature research shows that most children with autism have obvious gastrointestinal symptoms such as diarrhea, constipation, esophageal reflux, abdominal pain, flatulence, food intolerance, fecal stench, and so on.^[26]^ The degree of the disease is positively correlated. In 1980 by American informed Association published by the diagnostic and statistical manual of mental illness—third edition for the first time to set up the Infantile Autism (Infantile Autism) in the diagnosis of, but for the description of the core symptoms are fuzzy, to be classified as “comprehensive development disorder” (pervasive developmental disorders) category. In later revisions, the 3 core symptoms of autism have been identified: problems with social interaction, verbal and nonverbal development, repetitive behaviors, and abnormal interests. In 2013, the latest of the diagnostic and statistical manual of mental illness—fifth edition, for the first time put forward the concept of ASD, classified as “neurodevelopmental disorders” category, the core symptoms in the “language barrier” a removal, keep 2, at the same time stressed the onset age shall be quoted in infancy or early childhood,^[27]^ it also laid a solid foundation for the subsequent research of ASD. There is no specific treatment for this disease at present, mainly education and training.^[28]^ to take care of children with ASD, parents need to learn new skills or adjust the way of life, so as to produce economic and mental pressure, making the quality of life^[29,30]^ a big drop in social development. Traditional Chinese medicine treatment has a certain effect on the improvement of the symptoms of the disease. The treatment methods are mainly oral Chinese medicine and acupuncture, but children are often not easy to cooperate. As a safe and effective green therapy, massage is easy to be accepted by children.

This review aims to systematically review all randomized controlled trials (RCTs) to assess the effectiveness and safety of massage treatment for patients with autism spectrum disorders.

## 2 Materials and methods

This systematic review protocol has been registered on OSF on October 25, 2020 (Registration number: DOI 10.17605/OSF.IO/F796E). The protocol follows the Cochrane Handbook for Systematic Reviews of Interventions and the Preferred Reporting Items for Systematic Reviews and Meta-Analysis Protocol (PRISMA-P) statement guidelines.^[31]^ We will describe the changes in our full review if needed.

## 3 Inclusion criteria for study selection

### 3.1 Type of studies

This review will include clinical RCTs of clinical massage therapy for ASD patients without any language or publication status restrictions. Non-RCTs, quasi-RCTs, case series, case reports, crossover studies, uncontrolled trials, and laboratory studies will not be included.

### 3.2 Type of participants

Participants who were diagnosed with ASD according to related guidelines or consensus are included in this review regardless of their age, race, and gender.

### 3.3 Type of interventions

Interventions will include any type of clinically performed massage for improvement of autism spectrum disorders. This will include Chinese Massage, Japanese Massage, Thai Massage, Swedish Massage, Tuina, Shiatsu, Remedial Massage, General Massage, Acupressure, Reflexology, Manual Lymphatic Drainage. Studies of ASD combined with other interventions such as acupuncture, herbal medicines, qigong, and yoga will be considered for exclusion.

Control: no intervention, treatments other than massage (e.g., usual or standard care, placebo, wait-list controls).

### 3.4 Type of outcome measures

#### 3.4.1 Main outcome(s)

The primary outcome at the end of treatment or at maximal follow-up is the clinical effective rate, which is categorized as cure, markedly effective, effective, or ineffective according to clinical symptoms, CARS scale, and ABC scale, etc.

#### 3.4.2 Additional outcome(s)

The secondary outcomes will include symptom scores (stomachache, stomach distention, and belching, etc), Questionnaires, such as CGI-S, CGI-I, ADI-R, ABC-T, CBCL, SRS, SNAP-IV, etc.

## 4 Search methods for the identification of studies

### 4.1 Electronic searches

We will search the following electronic bibliographic databases for relevant trials:

CNKI (China National Knowledge Infrastructure Database, from 1979 to present;
Wanfang Database (from 1990 to present);
Pubmed Database (from 2000 to present);
CENTRAL (Cochrane Central Register of Controlled Trials, from 2000 to present);
CINAHL (Cumulative Index of Nursing and Allied Health Literature, from 1937 to present);
EMBASE (Excerpta Medica database, from 1947 to present);
Ovid MEDLINE ALL (Ovid Medical Literature Analysis and Retrieval System Online, from 1946 to present);
In addition, Clinical trial registries, like the Chinese Clinical Trial Registry (ChiCTR), the Netherlands National Trial Register (NTR) and ClinicalTrials.gov, will be searched for ongoing trials with unpublished data.

There will be no language restrictions.

### 4.2 Data collection and analysis

#### 4.2.1 Study identification

We will use EndNote X9 software to manage the records of searched electronic databases. The initial selection will involve scanning of the titles and abstracts of the retrieved studies. The full text of relevant studies will then be reviewed for study inclusion, in accordance with the inclusion criteria, by 2 authors (K-LZ and SD). Potentially relevant articles will be reviewed independently by 2 authors to determine if they meet the prespecified criteria. Any disagreement between authors will be resolved by consensus with a third author. The study selection procedure will follow and be recorded in the PRISMA flow chart. All the evidence will be assessed by The Grading of Recommendations Assessment, Development, and Evaluation (GRADE).

#### 4.2.2 Data extraction and management

According to the inclusion criteria, a standard data collection form will be made before data extraction. The following data will be extracted by 2 authors (K-LZ and SD):

*General information*: research identification, publication year, the title of the study, first author;
*Study methods*: study design, sample size, randomization method, allocation concealment, blinding, incomplete report or selecting report, other sources of bias;
*Participants*: inclusion and exclusion criteria;
*Intervention*: motion details, treatment duration, and frequency;
*Control*: type of control methods, motion details, treatment duration, and frequency;
*Outcomes*: included outcome measures.

#### 4.2.3 Risk of bias assessment

The risk of bias in included studies will be assessed independently by 2 reviewers (K-LZ and SD) using the Cochrane Risk of Bias Tool, with any disagreements resolved by consensus or by discussion with a third reviewer. All judgments will be fully described, and the conclusions will be presented in the Risk of Bias figures and will be incorporated into the interpretation of review findings, by means of sensitivity analysis. The risk of bias of each domain will be graded as adequate, unclear, or inadequate. We intend to use the concealment of allocation grading in investigation of any heterogeneity and in sensitivity analysis. Other aspects of study quality including the extent of blinding (if appropriate), losses to follow-up, noncompliance, whether the outcome assessment was standardized, and whether an intention to treat analysis was undertaken, will be presented in the risk of bias table describing the included studies and will provide a context for discussing the reliability of the results.

#### 4.2.4 Data analysis

We will use Stata Software [Computer program] (Version 15.1) to process the meta-analysis. Weighted mean difference will be used for continuous variable data, and the combined statistical effects of these 2 are combined. The *χ*^2^ test will be adopted to analyze whether there is heterogeneity in each of the included research questions. I^2^ > 50% is a criterion for significant judgment. The fixed effect model is adopted if I^2^ ≤ 50%, which is considered to have homogeneity between the studies. The random effect model is adopted if I^2^ > 50%, which is considered to have heterogeneity among the studies. The effect size is expressed as 95% confidence interval, and *P* < .05 is considered to be statistically significant.

##### 4.2.4.1 Sensitivity analyses

Heterogeneity may be due to the presence of 1 or more outlier studies with results that conflict with the rest of the studies. We will perform sensitivity analyses excluding outlier studies. In addition, we plan to perform sensitivity analysis to explore the influence of trial quality on effect estimates. The quality components of methodology include adequacy of generation of allocation sequence, concealment of allocation, and the use of intention-to-treat analysis.

##### 4.2.4.2 Meta-regression analyses

If data permits, we will perform the meta-regression analyses.

#### 4.2.5 Publication bias

If sufficient number of trials (more than 10 trials) are found, we will generate funnel plots (effect size against standard error) to investigate publication bias.

#### 4.2.6 Ethics and dissemination

The data used in this systematic review will be collected from published studies. Based on this, the study does not require ethical approval.

## Acknowledgments

The authors thank all the researchers in our working group.

## Author contributions

SG, KLZ, XC contributed on methodology and are the guarantors of the review.

KLZ, SD, SG and XNX contributed on data search, analysis, and statistics.

**Conceptualization:** Kelin Zhou, Jingyi Yang, Zhenbo Liu, Xia Cui.

**Data curation:** Ke-Lin Zhou, Shuo Dong, Zhen-Bo Liu, Jing-Yi Yang, Sheng Guo.

**Formal analysis:** Sheng Guo.

**Funding acquisition:** Xia Cui.

**Methodology:** Sheng Guo, Ke-Lin Zhou, Xia Cui.

**Resources:** Kelin Zhou, Shuo Dong, Xiaona Xue, Zhen-Bo Liu, Pei-dong Wei.

**Software:** Kelin Zhou, Shuo Dong, Jingyi Yang, Zhen-Bo Liu.

**Supervision:** Guo-Bing Fu, Jing-Yi Yang.

**Validation:** Xiaona Xue, Peidong Wei, Zhenbo Liu.

**Visualization:** Peidong Wei.

**Writing – original draft:** Ke-Lin Zhou, Shuo Dong.

**Writing – review & editing:** Guo-Bing Fu, Sheng Guo, Xia Cui.
